# Acute exacerbations of chronic obstructive pulmonary disease in a cohort of Chinese never smokers goes along with decreased risks of recurrent acute exacerbation, emphysema and comorbidity of lung cancer as well as decreased levels of circulating eosinophils and basophils

**DOI:** 10.3389/fmed.2022.907893

**Published:** 2022-08-10

**Authors:** Guangdong Wang, Aiping Ma, Liang Zhang, Jiaxi Guo, Qun Liu, Frank Petersen, Zhanxiang Wang, Xinhua Yu

**Affiliations:** ^1^Department of Respiratory and Critical Medicine, The First Affiliated Hospital of Xiamen University, Xiamen, China; ^2^Priority Area Chronic Lung Diseases, Research Center Borstel, Airway Research Center North, Member of the German Center for Lung Research (DZL), Borstel, Germany; ^3^Department of Neurosurgery, The First Affiliated Hospital of Xiamen University, Xiamen, China

**Keywords:** chronic obstructive pulmoanry disease, acute exacerbation, smoking, emphysema, lung cancer, hypercoagulation, eosinophils, basophils

## Abstract

Acute exacerbations show a significant impact on disease morbidity and mortality in chronic obstructive pulmonary disease (COPD). In contrast to stable COPD, the association of smoking status with clinical and laboratory characteristics in patients with acute exacerbations of COPD (AECOPD) has not been well studied. In this retrospective study, we compared never smokers and ever smokers on their demographic, clinical, and laboratory characteristics in a Chinese clinical cohort of AECOPD. In this cohort comprising 1,034 consecutive patients with AECOPD, never smokers were older (75 vs 70.5 years, *p^adjusted^* < 0.001) and had a higher body mass index than smokers (21.1 ± 4.0 vs 20.3 ± 3.4, *p^adjusted^* = 0.028). Furthermore, never smokers showed a decreased risk of recurrent acute exacerbation (13.0 vs 21.8%, *p^adjusted^* = 0.029), a lower risk of development of emphysema (77.8 vs 89.1%, *p^adjusted^* < 0.001), a lower prevalence of the co-morbidity of lung cancer (0.5 vs 6.6%, *p^adjusted^* < 0.001), lower levels of circulating eosinophils (EO; 0.04 × 10^9^/L vs 0.10 × 10^9^/L, *p^adjusted^* = 0.007) and basophils (BA; 0.02 × 10^9^/L vs 0.03 × 10^9^/L, *p^adjusted^* = 0.019), and a higher plasma levels of D-dimer (0.62 μg/ml vs 0.51 μg/ml, *p^adjusted^* = 0.02). Furthermore, multivariate logistic regression analysis identified several risk factor for the recurrent acute exacerbation, such as smoking [odds ratio (*OR*) = 1.84, 95% *CI*: 1.03–3.40, *p* = 0.044], urban residential area (*OR* = 1.43, 95% *CI*: 1.01–2.05, *p* = 0.045), and the presence of emphysema (*OR* = 2.31, 95% *CI*: 1.25–4.69, *p* = 0.012). In conclusion, this study demonstrates that the smoking status of patients is associated with recurrent acute exacerbations, emphysema, lung cancer, and levels of circulating EO and BA in AECOPD. Identification of cigarette smoking as a risk factor for recurrent acute exacerbation supports behavioral intervention of smoking cessation in the management of patients with AECOPD.

## Introduction

Chronic obstructive pulmonary disease (COPD) is a progressive respiratory disease featured by obstruction of the airways leading to a largely irreversible airflow limitation (1). Pathological hallmarks of COPD are persistent pulmonary inflammation, obstructive bronchiolitis, and emphysema (1). According to the World Health Organization’s estimates, COPD is the third leading cause of death worldwide, causing 3.23 million deaths in 2019 (2). COPD-caused mortality is largely related to acute exacerbation which is defined as worsening of the patient’s condition from the stable state and requiring additional care and therapy (3). For example, Soler-Cataluna et al. reported that patients with recurrent acute exacerbations have the highest mortality rate, with a risk of death 4.3 times higher than patients requiring no hospital management (4). Besides medical care and disease mortality, acute exacerbations of COPD (AECOPD) show an impact on multiple other clinical features, such as systemic inflammation, co-morbidities, and lung function, as well as laboratory phenotypes such as hematological parameters and coagulation markers (5, 6).

Although cigarette smoking is the main cause of COPD, approximately 30% patients with COPD are never smokers (7, 8). It has been shown in many studies that the two groups of patients with COPD differ in demographic and clinical features (7–12), suggesting that they are different in disease etiology and pathogenesis and thus should not be managed in the same way. In contrast to stable COPD, the association of smoking status with clinical and laboratory phenotypes in patients with AECOPD has only been investigated in few studies with small sample sizes (13, 14). In the present study, we aimed to determine the effect of smoking status on demographic, clinical, and laboratory characteristics of AECOPD in a large clinical cohort consisting of 1,034 patients.

## Materials and methods

### Patients

All patients with AECOPD were consecutively recruited between January 2015 and September 2021 in the First Affiliated Hospital of Xiamen University, Fujian, China. COPD was diagnosed according to the European Respiratory Society/American Thoracic Society guidelines (15). The post-bronchodilator fixed criteria forced expiratory volume in 1 s (FEV1)/forced vital capacity (FVC) < 0.7 was applied to define COPD (15). Pulmonary emphysema was diagnosed when the presence of low-attenuation areas below –950 Hounsfield Units was larger than 6% on computed tomography scans (16). According to the Global Initiative for Chronic Obstructive Lung Disease executive summary, AECOPD was defined as an event that a patient previously diagnosed with COPD experienced an acute worsening of respiratory symptoms demanding additional therapy (17). All AECOPD patients enrolled in this study had either moderate or severe exacerbation and were hospitalized and treated with inhaled corticosteroid. Recurrent acute exacerbation defined as ≥2 acute exacerbations in 12 months was evaluated for patients recruited before May 31, 2021 and followed until May 31, 2022. This study was performed in accordance with the 1964 Helsinki Declaration and its later amendments or comparable ethical standards. The ethics approval of the study was obtained from the Ethical Committee of the Clinical Research Ethics Committee of the First Affiliated Hospital of Xiamen University (No. 2021064). Since this retrospective study contains anonymous patient information, informed consent was waived by the institutional review board.

### Data collection

Demographic characteristics such as age, sex, residential location, education levels, smoking status, and body mass index (BMI), as well as clinical characteristics such as FEV1/FVC ratio, FEV1%pred, emphysema, and acute exacerbations, were retrieved from medical records for all patients and control subject if applicable. Common co-morbidities such as hypertension, heart failure, atrial fibrillation, coronary artery disease, cardiac valve disease, diabetes, hyperlipidemia, hyperuricemia, hypoproteinemia, bronchiectasis, asthma, tuberculosis, chronic rhinitis, lung cancer, and other cancers were also retrieved from the medical record. Peripheral venous blood was collected from all patients in the morning at the first day of admission and processed immediately. Hematological parameters were determined using Sysmex XN-9000 (XN, Sysmex, Kobe, Japan). Counts of white blood cells (WBCs), neutrophils (NE), eosinophils (EO), basophils (BA), lymphocytes (LYM), and monocytes (MO) were used for analysis. In addition, coagulation markers such as prothrombin time (PT), activated partial thromboplastin time (APTT), thrombin time (TT), fibrinogen (FIB), and D-dimer levels were measured by using CS-5100 automated coagulation (Sysmex, Kobe, Japan) for each patients at the time of admission were also retrieved from the medical record for the analysis. For patients with multiple exacerbations, data obtained from the first acute exacerbation were utilized for the analysis.

### Statistical analyses

All statistical analyses were performed with R software (R, version 4.1.1). The Kolmogorov–Smirnov normality test was performed to examine if variables are normally distributed. Quantitative variables which are not normally distributed were shown as median [25% quartile (Q1) – 75% quartile (Q3)], while variables with normal distribution were shown as mean ± standard deviation (*SD*). To determine statistical significance, quantitative data in normal distribution were compared using the student *t*-test; otherwise, the Mann–Whitney *U* test was used. For qualitative variables, data were expressed as numbers (percentages), and *p* values were determined using the chi-squared test or Fisher’s exact test. All *p* values were adjusted for multiple tests to prevent type I error using the method Benjamini–Hochberg (BH) and an adjusted *p* value less than 0.05 was considered as statistically significant (18). Multivariable logistic regression was performed to determine risk factors for recurrent acute exacerbation and to calculate Odds ratios (*OR*), 95% *CIs*, and *p* values. The generalized variance inflation factor was used to detect the presence of linear relationships between two or more independent variables.

## Results

### Demographic, clinical, and laboratory characteristics of patients with acute exacerbations of chronic obstructive pulmonary disease

In total, 1,034 consecutive patients with AECOPD were recruited for this study (Table 1). The mean age of patients with AECOPD was 71.1 ± 9.6 years (mean ± *SD*), and the majority of patients (92.3%) were men. Approximately half of the patients (52.2%) came from urban communities while all others originated from either suburban or rural areas. When patients were categorized according to educational levels, only one-fifth of cases received high school education. Of the 1,034 patients with AECOPD, 200 were never smokers, and 834 were ever smokers, such as 489 current smokers and 345 former smokers, with a median smoking history of 40 pack-years. In total, 86.47% of patients with AECOPD developed emphysema, and 87.0% of acute exacerbations were associated with infection. The median hospitalization time in all AECOPD patients was 7 days. Among 977 patients recruited before May 31, 2021, 196 patients (20.06%) were featured by recurrent acute exacerbation. The frequencies of common comorbidities, namely hypertension, heart failure, coronary artery disease, atrial fibrillation, cardiac valve disease, diabetes, hyperlipidemia, hyperuricemia, hypoproteinemia, bronchiectasis, asthma, tuberculosis, chronic rhinitis, lung cancer, and other cancers were 37.1, 8.5, 10.6, 5.4, 5.7, 9.1, 7.4, 6.0, 11.1, 6.6, 9.8, 13.6, 6.9, 5.4, and 4.8%, respectively. Besides above-mentioned demographic and clinical characteristics, laboratory phenotypes such as hematological parameters and coagulation markers in patients with AECOPD were summarized in Table 1.

**TABLE 1:** Demographic, clinical, and laboratory features of patients with acute exacerbations of chronic obstructive pulmonary disease (AECOPD).

		AECOPD (*n* = 1,034)
Age**		71 (64–78)
Gender	Male	954 (92.3%)
	Female	80 (7.7%)
Residential locations	Urban area	540 (52.2%)
	Suburban area	46 (4.5%)
	Rural area	448 (43.3%)
Education^#^	Low (≤9 years)	833 (80.6%)
	High (>9 years)	200 (19.4%)
Smoking status	Never-smoker	200 (19.3%)
	Former smoker	345 (33.4%)
	Current smoker	489 (47.3%)
Smoking history (pack-years)**		40 (20–60)
BMI**		20 (17.8–22.68)
Clinical features	FEV1/FVC*^$^	52.3 ± 11.0
	FEV1%pred**^$^	45.0 (33.0–58.0)
	Emphysema	86.5% (894/1,034)
	Duration of illness (Days) **	7 (6–10)
	Acute exacerbations associated with infection	87.0% (900/1,034)
	Recurrent acute exacerbations ^§^	20.1% (196/977)
Common comorbidities	Hypertension	37.1% (384/1,034)
	Heart failure	8.5% (88/1,034)
	Coronary artery disease	10.6% (110/1,034)
	Atrial fibrillation	5.4% (56/1,034)
	Cardiac valve disease	5.7% (59/1,034)
	Diabetes	9.1% (94/1,034)
	Hyperlipidemia	7.4% (76/1,034)
	Hyperuricemia	6.0% (62/1,034)
	Hypoproteinemia	11.1% (115/1,034)
	Bronchiectasis	6.6% (68/1,034)
	Asthma	9.8% (101/1,034)
	Tuberculosis	13.6% (141/1,034)
	Chronic rhinitis	6.9% (71/1,034)
	Lung cancer	5.4% (56/1,034)
	Other cancers	4.8% (50/1,034)
Hematological parameters**	WBC (10^9^/L)	8.15 (6.37–10.88)
	NE (10^9^/L)	5.73 (4.15–8.25)
	LYM (10^9^/L)	1.30 (0.87–1.80)
	MO (10^9^/L)	0.66 (0.49–0.90)
	BA (10^9^/L)	0.03 (0.01–0.05)
	EO (10^9^/L)	0.09 (0.01–0.2)
Coagulation markers**	PT (s; 9–15)	13.40 (12.30–14.20)
	APTT (s; 17–37)	36.45 (28.23–41.50)
	TT (s; 14–21)	15.70 (15.00–16.60)
	FIB (g/L; 1.8–3.5)	4.32 (3.27–5.47)
	D-D (μg/ml; 0–0.5)	0.50 (0.31–1.06)

Quantitative data are presented as * Mean ± SD or ** Median (Q1–Q3). ^§^ Data from 977 patients recruited before 31th May 2021. ^#^Lack of information of one patient. ^$^Lack of information of patients who were unable to perform the pulmonary function tests. Reference ranges for coagulation parameters are shown.

### Association of smoking status with demographic and clinical characteristics in patients with acute exacerbations of chronic obstructive pulmonary disease

We first examined the association of the smoking status with demographical and clinical characteristics of patients with AECOPD. Of note, the median age in 200 never smokers was 4.5 years higher than that in 834 ever smokers (75 vs 70.5, *p^adjusted^* < 0.001). Smoking status was also significantly associated with gender and body weight, where never smokers comprised few men (70.0 vs 97.6%, *p^adjusted^* < 0.001) and showed a higher BMI (20.9 vs 19.8, *p^adjusted^* = 0.028) than ever smokers. However, the two subgroups did neither differ significantly in their educational levels nor in their residential location. Regarding clinical characteristics, ever smokers showed an increased risk of emphysema (89.1 vs 77.8%, *OR* = 2.37, 95% *CI*: 1.60–3.52, *p^adjusted^* < 0.0001) and a higher recurrent acute exacerbation (21.8 vs 13.0%, *OR* = 1.81, 95% *CI*: 1.15–2.84, *p^adjusted^* = 0.029) than never smokers, but the two groups were comparable in terms of lung function and duration of hospitalization (Table 2).

**TABLE 2:** Association of smoking status with demographic and clinical characteristics in patients with AECOPD.

		Never-smoker (*n* = 200)	Ever-smoker (*n* = 834)	OR (95% CI)	*P* adj**
Age (Years)*		75 (67–81)	70.5 (64–84)	–	<0.001
Gender	Male	140 (70.0%)	814 (97.6%)	17.44 (10.20–29.84)	<0.001
	Female	60 (30.0%)	20 (2.4%)		
Residential locations	Urban area	109 (54.5%)	431 (51.7%)	–	0.901
	Suburban area	9 (4.5%)	37 (4.4%)		
	Rural area	82 (41.0%)	366 (43.9%)		
Education	Low	38 (19.0%)	162 (19.5%)	1.02 (0.70–1.52)	0.901
	High	162 (81.0%)	671 (80.6%)		
BMI*		20.9 (18.0–23.7)	19.8 (17.7–22.6)	–	0.028
FEV1/FVC*		54.0 (47.0–61.0)	51.6 (44.0–61.0)	–	0.256
FEV1%pred*		45.0 (33.5–56.5)	45.0 (33.0–59.0)	–	0.901
Duration of illness (Days)*		8 (6–10)	7 (6–10)	–	0.819
Emphysema		154 (77.8%)	740 (89.1%)	2.37 (1.60–3.52)	<0.001
Acute exacerbation associated with infection		175 (87.5%)	725 (86.9%)	0.95 (0.60–1.51)	0.901
Recurrent acute exacerbations^§^		25 (13.0%)	171 (21.8%)	1.81 (1.15–2.84)	0.029

*Data are presented as median (Q1–Q3). ^§^ Data from 977 patients recruited before 31th May 2021. ** *p* values are adjusted for multiple tests using the method Benjamini–Hochberg.

### Association of smoking status with common co-morbidities and coagulation markers in patients with acute exacerbations of chronic obstructive pulmonary disease

Next, we investigated whether smoking status is associated with common co-morbidities in AECOPD. The most significant difference between ever smokers and never smokers was observed with regard to lung cancer, where the prevalence of patients with this co-morbidity was more than 10 times higher in smokers than that in never smokers (6.6 vs 0.5%, *OR* = 14.1, 95% *CI*: 1.93–102.15, *p^adjusted^* < 0.001). By contrast, such a difference was not observed in the proportion of patients showing other cancers (5.0 vs 4.0%, *p* = 0.54). In addition, frequencies of none of the other comorbidities were comparable between ever smokers and never smokers, with only an exception of a tendency of an increased risk of hypertension in never smokers compared to ever smokers (39.5 vs 32.3%, *p* = 0.051, *p^adjusted^* = 0.175; Table 3).

**TABLE 3:** Association of smoking status with comorbidities in patients with AECOPD.

	Never-smoker (*n* = 200)	Ever-smoker (*n* = 834)	OR (95% CI)	*P* adj*
Hypertension	79 (39.5%)	269 (32.3%)	0.73 (0.53–1.00)	0.175
Heart failure	19 (9.5%)	69 (8.3%)	0.86 (0.50–1.46)	0.819
Atrial fibrillation	14 (7.0%)	42 (5.0%)	0.71 (0.38–1.32)	0.526
Coronary artery disease	25 (12.5%)	85 (10.2%)	0.79 (0.49–1.28)	0.633
Cardiac valve disease	14 (7.0%)	55 (6.6%)	0.93 (0.51–1.72)	0.901
Diabetes	20 (10.0%)	74 (8.9%)	0.88 (0.52–1.47)	0.819
Hyperlipidemia	13 (6.5%)	63 (7.6%)	1.17 (0.63–2.18)	0.819
Hyperuricemia	11 (5.5%)	51 (6.1%)	1.12 (0.57–2.19)	0.901
Hypoproteinemia	25 (12.5%)	90 (10.8%)	0.85 (0.53–1.36)	0.819
Bronchiectasis	18 (9.0%)	50 (6.0%)	0.64 (0.36–1.13)	0.328
Asthma	19 (9.5%)	82 (9.8%)	1.04 (0.61–1.75)	0.901
Tuberculosis	25 (12.5%)	116 (13.9%)	1.13 (0.71–1.80)	0.819
Chronic rhinitis	10 (5.0%)	61 (7.3%)	1.49 (0.75–2.98)	0.503
Lung cancer	1 (0.5%)	55 (6.6%)	14.1 (1.93–102.15)	<0.001
Other cancers	8 (4.0%)	42 (5.0%)	1.27 (0.58–2.75)	0.819

* *p* values are adjusted for multiple tests using the method Benjamini–Hochberg.

### Association of smoking status with laboratory characteristics in patients with acute exacerbations of chronic obstructive pulmonary disease

It has been reported that AECOPD is featured by hypercoagulability and elevated levels of circulating WBCs (6); we next examined whether the smoking status is associated with hematological parameters and coagulation markers. As shown in Table 4, the count of EO was significantly higher in smokers than that in never smokers (0.10 × 10^9^/L vs 0.04 × 10^9^/L, *p^adjusted^* = 0.007). Compared with never smokers, smokers in patients with AECOP also showed higher levels of BA (0.03 × 10^9^/L vs 0.02 × 10^9^/L, *p^adjusted^* = 0.019). With regards to coagulation markers, never smokers showed comparable levels of PT, APTT, TT, and FIB as ever smokers. However, significantly higher levels of D-dimer were observed in never smokers as compared to ever smokers (0.62 vs 0.51 μg/ml, *p^adjusted^* = 0.02).

**TABLE 4:** Association of smoking status with laboratory characteristics in patients with AECOPD.

		Never-smoker (*n* = 200)	Ever-smoker (*n* = 834)	*P*-value	*P* adj
Hematological parameters*	WBC (10^9^/L)	8.09 (6.46–11.24)	8.16 (6.30–10.77)	0.399	0.703
	NE (10^9^/L)	5.86 (4.35–8.99)	5.70 (4.11–8.13)	0.173	0.400
	LYM (10^9^/L)	1.20 (0.79–1.66)	1.30 (0.90–1.81)	0.023	0.085
	MO (10^9^/L)	0.66 (0.45–0.89)	0.67 (0.50–0.90)	0.225	0.490
	BA (10^9^/L)	0.02 (0.01–0.04)	0.03 (0.01–0.05)	0.003	0.019
	EO (10^9^/L)	0.04 (0–0.19)	0.10 (0.02–0.20)	0.001	0.007
Coagulation markers*	PT (s)	13.50 (12.40–14.40)	13.30 (12.20–14.10)	0.082	0.279
	APTT (s)	34.70 (29.15–40.90)	36.80 (28.15–41.95)	0.165	0.426
	TT (s)	15.70 (15.10–16.50)	15.75 (15.00–16.60)	0.901	0.901
	FIB (g/L)	4.28 (3.38–5.33)	4.32 (3.25–5.53)	0.893	0.901
	D-D (ug/ml)	0.62 (0.36–1.38)	0.51 (0.29–1.01)	0.004	0.02

*Data are presented as median (Q1–Q3).

### Risk factors for recurrent acute exacerbations

Given that the above results showed that smoking status was associated with recurrent acute exacerbations, we next investigated whether cigarette smoking is an independent risk factor for this clinical phenotype. For this purpose, we performed a multivariate logistic regression analysis using age, gender, smoking status, smoking pack years, BMI, education levels, residential locations, emphysema, and infections variables. The multivariate logistic regression analysis showed that cigarette smoking was a risk factor for the recurrent acute exacerbation (*OR* = 1.84, 95% *CI*: 1.03–3.40, *p* = 0.044). Besides smoking status, urban residential area (*OR* = 1.43, 95% *CI*: 1.01–2.05, *p* = 0.045) and the presence of emphysema (*OR* = 2.31, 95% *CI*: 1.25–4.69, *p* = 0.012) were demonstrated to be risk factors for recurrent acute exacerbations (Table 5).

**TABLE 5:** A multivariable logistic regression analysis of risk factors for recurrent acute exacerbations.

Variables	GVIF	OR (95% CI)	*P*-value
Age*	1.05	0.99 (0.98–1.01)	0.411
Gender: (male vs female)	1.21	0.79 (0.38–1.72)	0.528
Smoking Status: (smokers vs never smokers)	1.52	1.84 (1.03–3.40)	0.044
Smoking history (pack-years)*	1.36	1.00 (0.99–1.01)	0.469
BMI*	1.02	1.00 (0.95–1.04)	0.862
Education: (≤9 years vs >9 years)	1.08	1.05 (0.75–1.63)	0.820
Residential location: (suburban area vs rural area)	1.11	0.66 (0.22–1.63)	0.411
Residential locations: (urban area vs rural area)		1.43 (1.01–2.05)	0.045
Emphysema: (yes vs no)	1.02	2.31 (1.25–4.69)	0.012
Acute exacerbation due to infection: (yes vs no)	1.02	1.67 (0.99–2.99)	0.067

*For quantitative variables, OR was calculated for one unit increase in age, smoking pack years and BMI. GVIF, generalized variance inflation factor.

## Discussion

In the current study, we investigated the effect of smoking status on the demographic, clinical, and laboratory features in a clinical cohort of 1,034 patients with AECOPD. We could demonstrate that never smokers show a higher age, a lower male-to-female ratio, a lower BMI, higher levels of D-dimer, lower levels of circulating EO and BA, as well as decreased risks for recurrent acute exacerbations, emphysema, and lung cancer.

To our knowledge, the current study for the first time demonstrates that smokers are more susceptible to recurrent acute exacerbations than never smokers in patients with AECOPD. Interestingly, in this cohort smoking status, but not smoking history, is an independent risk factor for recurrent acute exacerbation. The difference between smoking status and smoking history in their effect on recurrent acute exacerbations might be explained by the method used for the calculation of ORs. The OR of smoking status is calculated by comparing smokers with never smokers, while that of smoking history, a quantitative variable, is calculated for every increase of 1 pack-year. The demonstration of cigarette smoking as a risk factor for recurrent acute exacerbations is in accordance with previous findings. For example, Au et al. reported that smoking cessation is associated with a reduced risk of COPD exacerbations (19). Furthermore, Dusemund et al. reported that a reduction of second-hand smoke by legislated bans on smoking is associated with reduced rates of AECOPD (20). Recurrent acute exacerbations mainly occur in patients with severe COPD and accelerate disease progression and mortality (5). For example, in 2005 Soler-Cataluna et al. reported that severe exacerbations of COPD are independent risk factors of a poor prognosis and mortality increases with the frequency of severe exacerbations (4). Therefore, the intervention of cigarette smoking cessation may be useful for controlling the frequency of acute exacerbation of COPD and thus improving patient’s outcome.

Among all common co-morbidities, only lung cancer was significantly different distributed between never smokers and ever smokers, with a higher frequency of lung cancer in the latter group. Given that cigarette smoking is the major cause of both COPD and lung cancer (21), it is not surprising to observe that smokers in patients with AECOPD show more prevalent co-morbidity of lung cancer than never smokers. Another smoking status-associated clinical characteristic is emphysema, with a higher prevalence of emphysema in smokers than in non-smokers in AECOPD. This result is in line with findings from a very recent study, in which Ding et al. demonstrated that smokers in AECOPD exhibit more severe diffuse dysfunction than never smokers and cigarette smoking represents an independent risk factor for emphysema (13). In COPD, emphysema is a consequence of chronic inflammatory and consequent destructive processes. Inflammatory cells such as NE and macrophages produce proteinases and oxidants that cause damage to alveolar cells, as well as the extracellular matrix, leading to the development of emphysema (22). Cigarette smoke is able to accelerate this process by directly inducing airway inflammation and amplifying the inflammation *via* activating epithelial cells to release pro-inflammatory mediators (23).

It is well recognized that cigarette smoking leads to elevated levels of WBCs, such as granulocytes, MO, and LYM, in healthy subjects (24). Interestingly, the current study showed that only levels of EO and BA are higher in smokers than never smokers in patients with AECOPD. This finding suggests that the effect of cigarette smoking on different types of leukocytes might be condition-dependent, which needs to be further elucidated. Another laboratory characteristic associated with smoking status in patients with AECOPD is plasma levels of D-dimer. D-dimer has been suggested to be a reliable prognostic marker for both short-term and long-term survival in patients admitted for AECOPD (25) and a strong and independent risk factor for hospitalization and mortality (26). Interestingly, levels of D-dimer differed significantly between the two subgroups of AECOPD, where never smokers show higher levels of D-dimer than ever smokers. However, the two subgroups of AECOPD patients show no significant difference in disease severity in terms of spirometric measures. In this AECOPD cohort, never smokers were older and with a higher female-to-male ratio than smokers. Since it has been shown previously that increase in plasma D-dimer is dependent on age but not sex (27), the elevated levels of D-dimer in never smokers might be explained by the difference in age between the two subgroups of patients with AECOPD. However, this notion needs to be verified in future studies.

The current study has some limitations. First of all, admission bias might occur in this single-center retrospective study because all patients were recruited from one hospital. For example, COPD patients were likely to be admitted to other hospitals due to acute exacerbation, which may lead to underestimating recurrent AECOPD. Secondly, the lack of information on occupational exposures and the second-hand smoke in never smokers prevents the further investigation of subgroups of never smokers, e.g., exposure to second-hand tobacco smoke or biomass smoke in AECOPD. Thirdly, in China, only 3.2% women are ever regular smokers, while the prevalence of cigarette smoking in men (70%) is dramatically higher than that in women (3.2%) among the general population in China (28). The male dominance in smokers leads to a substantial difference in the male-to-female ratio between never smokers and ever smokers in patients with AECOPD, which needs to be taken into consideration when interpreting results. Finally, this retrospective study is also limited by a lack of information on survival data of patients and clinical characteristics measured between the diagnosis of COPD and AECOPD.

In summary, our results demonstrate that smoking status is associated with multiple demographic, clinical, and laboratory characteristics. Identification of cigarette smoking as an independent risk factor for recurrent acute exacerbations supports the behavioral intervention of smoking cessation in the management of patients with AECOPD.

## Data availability statement

The original contributions presented in this study are included in the article/supplementary material, further inquiries can be directed to the corresponding authors.

## Ethics statement

The studies involving human participants were reviewed and approved by the Ethical Committee of the Clinical Research Ethics Committee of the First Affiliated Hospital of Xiamen University. Written informed consent for participation was not required for this study in accordance with the national legislation and the institutional requirements.

## Author contributions

XY, ZW, and AM conceived and supervised the study. GW, AM, JG, and QL collected data. LZ analyzed the data. XY, FP, LZ, and AM wrote the manuscript. All authors have read and approved the final manuscript.

## Abbreviations

APTT: activated partial thromboplastin time
AECOPD: acute exacerbations of chronic obstructive pulmonary disease
BMI: body mass index
BA: basophils
CI: confidence interval
COPD: chronic obstructive pulmonary disease
EO: eosinophils
FIB: fibrinogen
FEV1: forced expiratory volume in 1 s
FVC: forced vital capacity
GOLD: Global Initiative for Chronic Obstructive Lung Disease
LYM: lymphocytes
MO: monocytes
NE: neutrophils
OR: odds ratios
PT: prothrombin time
SD: standard deviation
TT: thrombin time
VIF: variance inflation factor
WBC: white blood cells.
